# Refractoriness of hepatitis C virus internal ribosome entry site to processing by Dicer in vivo

**DOI:** 10.1186/1477-5751-8-8

**Published:** 2009-08-13

**Authors:** Dominique L Ouellet, Isabelle Plante, Vincent Boissonneault, Cherifa Ayari, Patrick Provost

**Affiliations:** 1 Centre de Recherche en Rhumatologie et Immunologie, CHUL Research Center/CHUQ, 2705 Blvd Laurier, Quebec, QC, G1V 4G2, Canada; 2 Faculty of Medicine, Université Laval, Quebec, QC, G1V 0A6, Canada

## Abstract

**Background:**

Hepatitis C virus (HCV) is a positive-strand RNA virus harboring a highly structured internal ribosome entry site (IRES) in the 5' nontranslated region of its genome. Important for initiating translation of viral RNAs into proteins, the HCV IRES is composed of RNA structures reminiscent of microRNA precursors that may be targeted by the host RNA silencing machinery.

**Results:**

We report that HCV IRES can be recognized and processed into small RNAs by the human ribonuclease Dicer in vitro. Furthermore, we identify domains II, III and VI of HCV IRES as potential substrates for Dicer in vitro. However, maintenance of the functional integrity of the HCV IRES in response to Dicer overexpression suggests that the structure of the HCV IRES abrogates its processing by Dicer in vivo.

**Conclusion:**

Our results suggest that the HCV IRES may have evolved to adopt a structure or a cellular context that is refractory to Dicer processing, which may contribute to viral escape of the host RNA silencing machinery.

## Background

Hepatitis C virus (HCV), a member of the *Flaviviridae *family, is a positive-strand RNA virus that establishes a persistent infection in the liver, leading to the development of chronic hepatitis, liver cirrhosis, and hepatocellular carcinoma [1]. HCV is one of the main causes of liver-related morbidity and mortality [2]. Its ~9,6-kilobase (kb) RNA genome, which is flanked at both termini by conserved, highly structured untranslated regions (UTRs), encodes a polyprotein processed by host and viral proteases to produce the structural (core, E1, E2-p7) and non-structural (NS2, NS3, NS4A, NS4B, NS5A, NS5B) proteins of the virus [3,4]. Located in its 5'UTR, the internal ribosome entry site (IRES) of HCV essentially controls translation initiation [5-8] in a process involving cellular [9] as well as viral [10-14] proteins. The HCV IRES contains several double-stranded RNA (dsRNA) regions forming stem-bulge-loop structures [15,16] analogous to that of microRNA precursors (pre-miRNAs).

Known to originate from Drosha processing of primary miRNAs (pri-miRNAs) in the nucleus [17], pre-miRNAs are the endogenous substrates of the ribonuclease III (RNase III) Dicer into the cytoplasm. Involved in the microRNA (miRNA)-guided RNA silencing pathway, Dicer converts pre-miRNAs into ~21 to 23-nucleotide (nt) RNA guide sequences [18,19], referred to as miRNAs. These short regulatory RNAs initially mediate translational repression or cleavage of specific messenger RNA (mRNA) targets [20,21]. RNA of exogenous origin, such as viruses, may also serve as substrates for Dicer. In virus-infected plants, antisense viral RNAs of ~25-nt were detected [22] and found to originate from viral dsRNA processing by Dicer, or DICER-like 1 in *Arabidopsis *[23]. More recently, human viruses such as Epstein-Barr virus (EBV) [24], Kaposi's sarcoma-associated herpesvirus (KSHV or HHV-8), human cytomegalovirus (HCMV) [25,26] and human immunodeficiency virus type 1 (HIV-1) [27-29] were reported to be a source of miRNAs. Conversely, a number of viruses have been shown to counteract miRNA-guided RNA silencing through the generation of suppressors of RNA silencing [30]. Examples include the E3L protein of vaccinia virus, NS1 protein of influenza virus [31], B2 protein of flock house virus (FHV) [32], non-structural proteins of La Crosse virus (LACV) [33] and, more recently, HCV structural core [34,35] and E2 [36] proteins that act as suppressors of Dicer and Argonaute 2 (Ago2), respectively.

As for the relationship between HCV and RNA silencing processes, it appears to be more complex than previously thought. Initial studies reported that small interfering RNAs (siRNAs) [37-39] and short hairpin RNAs (shRNAs) [40,41] directed against HCV were effective in reducing viral replication in human liver cells. On the other hand, a liver-specific miRNA derived from Dicer, miR-122, was shown to facilitate HCV replication through an unknown mechanism involving the recognition of a specific sequence in the 5'UTR of the viral RNA [42]. These observations support the notion that the HCV RNA is accessible to components of the miRNA-guided RNA silencing machinery, such as Dicer, and thus susceptible to be processed into smaller RNAs.

In the present study, we report that HCV does not contain inhibitors of RNA silencing among its non-structural proteins and that Dicer remains functional in 9–13 cells harboring HCV subgenomic replicon. Conversely, the HCV IRES and its isolated domains II, III and VI are prone to Dicer cleavage in vitro. However, maintenance of its functional integrity in response to Dicer overexpression in vivo suggests that the HCV IRES may have evolved to adopt a structure refractory to Dicer processing or that the accessibility of HCV IRES of Dicer is limited in the intracellular environment.

## Results

### HCV has no effect on miRNA-guided RNA silencing

**Figure 1:**
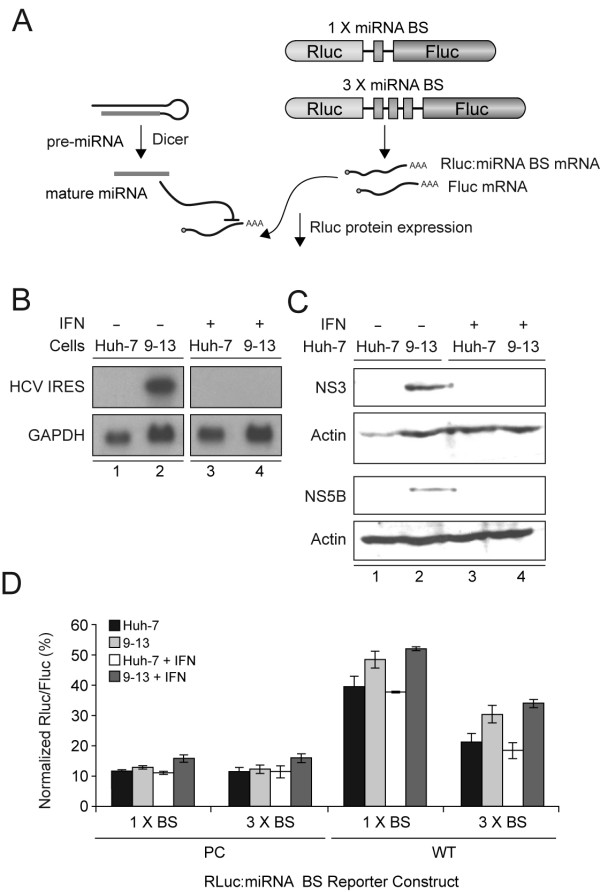
**miRNA-guided RNA silencing is not perturbed in cells harboring a subgenomic HCV replicon**. (A) Schematic representation of the experimental strategy and reporter gene constructs. (B) HCV RNA expression in Huh-7 or 9–13 cells harbouring a subgenomic HCV replicon, treated or not with 100 IU/ml of interferon α-2B (IFNα-2B), was documented by Northern blot using a DNA probe complementary to HCV *Internal ribosome entry site *(nt 1 to 341). GAPDH was used as a loading control. (C) HCV NS3 and NS5B protein expression Huh-7 or 9–13 cells, treated or not with 100 IU/ml of IFNα-2B, was documented by Western blot using anti-NS3 1B6 (first panel) and anti-NS5B 5B-3B1 (third panel) antibodies, respectively. Actin was used as a loading control (second and fourth panels). (D) Huh-7 or 9–13 cells, treated or not with 100 IU/ml of IFNα-2B, were cotransfected using Lipofectamine 2000 with a Rluc:miRNA binding site construct, in which the Rluc reporter gene is coupled with 1 or 3 copies of perfectly complementary (PC) or natural wild-type (WT) binding sites (BS) for miR-328 (250 ng DNA), and a psiSTRIKE-based, pre-miR-328 expression construct (250 ng DNA). psiSTRIKE-Neg, which encodes a shRNA directed against a sequence deleted in the Rluc reporter mRNA, was used as a control. Results of Rluc activity were normalized with Fluc activity and expressed as a percentage of Rluc activity obtained with psiSTRIKE-Neg. Results are expressed as mean ± s.e.m. (n = 3 experiments, in duplicate).

In order to determine if HCV harbors non-structural proteins that could interfere with Dicer function in RNA silencing processes, we examined the efficiency of a natural Dicer substrate, i.e. a pre-miRNA, to induce RNA silencing in 9–13 cells harboring a subgenomic HCV replicon, as illustrated in Fig. 1A. First, expression of HCV RNA (see Fig. 1B, upper panel, lane 2) as well as that of NS3 (see Fig. 1C, first panel, lane 2) and NS5B (see Fig. 1C, third panel, lane 2) proteins was confirmed in 9–13 cells harboring a subgenomic HCV replicon. As expected, no HCV RNA (see Fig. 1B, upper panel, lane 1) or proteins (see Fig. 1C, first and third panels, lane 1) was detected in the host Huh-7 cell line. To assess the efficiency of RNA silencing, we utilized an adapted assay based on the regulation of Rluc reporter gene activity through expression of a natural Dicer substrate. In this assay, the imperfectly paired stem-loop structured pre-miR-328 is processed by Dicer into miR-328, which then induces silencing of a Rluc reporter gene coupled with 1 or 3 copies of a sequence perfectly complementary (PC) to miR-328 (see Fig. 1A) or that of its naturally occurring, wild-type (WT) binding site of imperfect complementarity, as described recently [43]. To verify the suitability of our approach, we assessed the effect of adenoviral VA1 RNA expression which has been shown to interfere with RNAi through a direct interaction with Dicer (see Additional file 1) [44]. Adenoviral VA1 RNA expression dose-dependently reduced the efficiency of RNA silencing, as expected. However, neither of PC or WT approaches could detect significant changes in the efficiency of RNA silencing that could be related to the presence of the subgenomic HCV replicon in 9–13 cells (see Fig. 1D). These results suggest that the function of Dicer and of the host miRNA-guided RNA silencing machinery is not perturbed by the HCV non-structural proteins.

We noted a slight intrinsic defect in the efficiency of RNA silencing mediated through recognition by miR-328 of its natural binding site of imperfect complementarity independent of the presence of HCV replicon (see Fig. 1D). These observations suggest that cell that may be deficient for at least one component of the RNAi pathway. It also suggests that cells grown continuously under pressure to keep the HCV replicon may have evolved slightly less efficient RNA silencing machinery. In vitro Dicer activity assays performed using Dicer immunoprecipitates incubated in the presence of human let-7a-3 pre-miRNA substrate suggest that the slight impairment of 9–13 cells in RNA silencing is unlikely due to an altered Dicer function (see Additional file 2).

We also studied Huh-7 and 9–13 cells pre-treated or not with interferon alpha-2B (IFNα-2B) [45,46]. Treatment with IFNα-2B effectively cured the 9–13 cells of the HCV replicon, as indicated by the loss of HCV RNA (see Fig. 1B, upper panel, lane 4) as well as of NS3 (see Fig. 1C, first panel, lane 4) and NS5B (see Fig. 1C, third panel, lane 4) proteins. However, miR-328 mediated silencing of Rluc expression via its WT binding sites was similar in cells harbouring or not the HCV replicon (Fig. 1D), indicating that the intrinsic differences in RNAi efficiency between the host cells are not related to HCV.

### Dicer binds and cleaves HCV IRES in vitro

**Figure 2:**
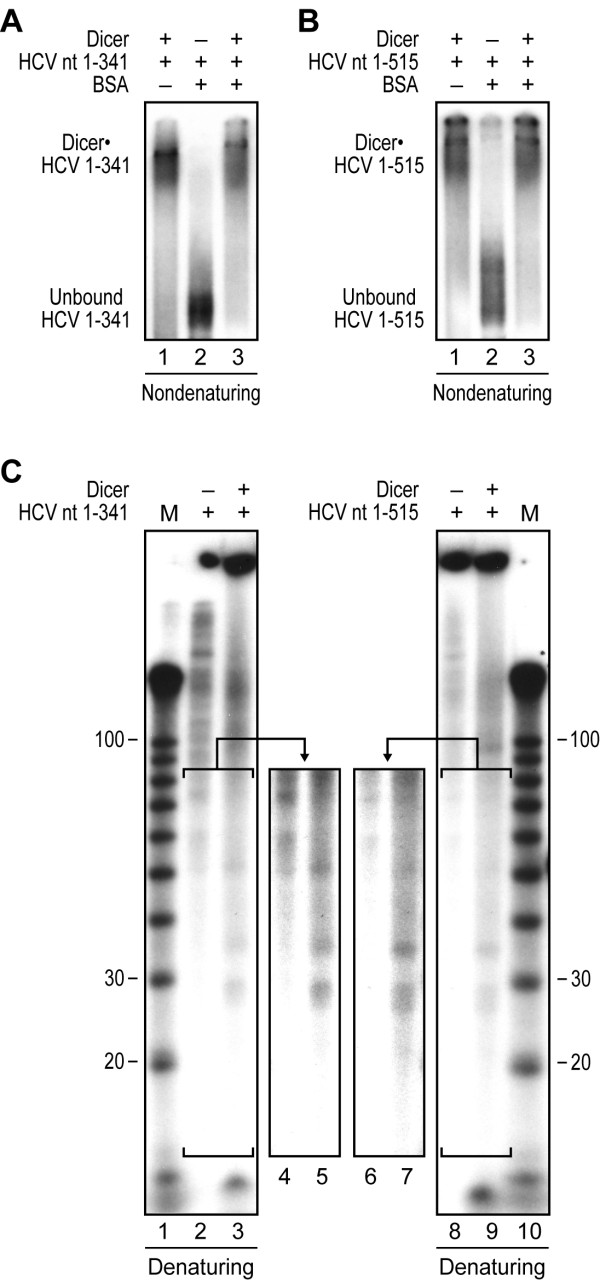
**Recombinant Dicer binds and cleaves HCV IRES in vitro**. (A-B) Electrophoretic mobility shift assays (EMSA) ^32^P-labeled HCV RNA nt 1-341 (A) or nt 1-515 (B) was incubated in the absence or presence of recombinant human Dicer (200 ng) and/or BSA (2 μg), and complex formation visualized by non-denaturing PAGE and autoradiography. (C-D) Dicer RNase activity assays. (C) ^32^P-labeled HCV RNA nt 1-341 (left panel) or nt 1-515 (right panel) was incubated in the absence (-) or presence (+) of recombinant human Dicer (200 ng), and HCV RNA processing monitored by denaturing PAGE and autoradiography. Lanes 4, 5, 6 and 7 represent higher numerical exposition of lanes 2, 3, 8 and 9 respectively. M, indicates a 10-nt RNA size marker.

The first 341 nt of the HCV genome forms a functional IRES unit, whereas the immediate downstream sequence (nt 341-515), which is dispensable for IRES function and referred to as the 5'core-coding sequence, contains two additional stem-loop structures, including domain VI. Together with the functionality of Dicer in 9–13 cells expressing the HCV subgenomic replicon, these observations prompted us to question whether Dicer could recognize and process the full-length HCV IRES RNA in vitro. Two ^32^P-labeled HCV IRES RNAs were prepared by in vitro transcription, i.e. HCV nt 1-341 and HCV nt 1-515, incubated in the absence or presence of recombinant human Dicer and/or BSA, and analyzed by electrophoretic mobility shift assay (EMSA). These experiments revealed that Dicer, but not BSA, reduced the mobility of the HCV IRES RNAs in nondenaturing gels (see Fig. 2A and 2B, lanes 1 and 3), an observation indicative of Dicer•HCV IRES RNA complex formation. Moreover, small amounts of ~21 to 28 nt RNA species were detected upon MgCl_2_-induced activation of Dicer RNase activity (see Fig. 2C, lanes 5 vs 4 and lanes 7 vs 6). The differences observed in small RNA length obtain in this assay could be a result from an asymmetric cleavage of Dicer as suggested for miR-TAR-5p and miR-TAR-3p processing from HIV TAR element [29]. Alternatively, it may be related to an imperfect folding of the HCV RNAs transcribed in vitro. However, the presence of a faint band corresponding to a ~22 nt RNA species (see Fig. 2C, lane 7) suggests that domain VI, which is included in the HCV IRES nt 1-515, but not in the HCV IRES nt 1-341 form, may represent a substrate for Dicer under these conditions.

### HCV domains II, III and VI are prone to Dicer processing in vitro

**Figure 3:**
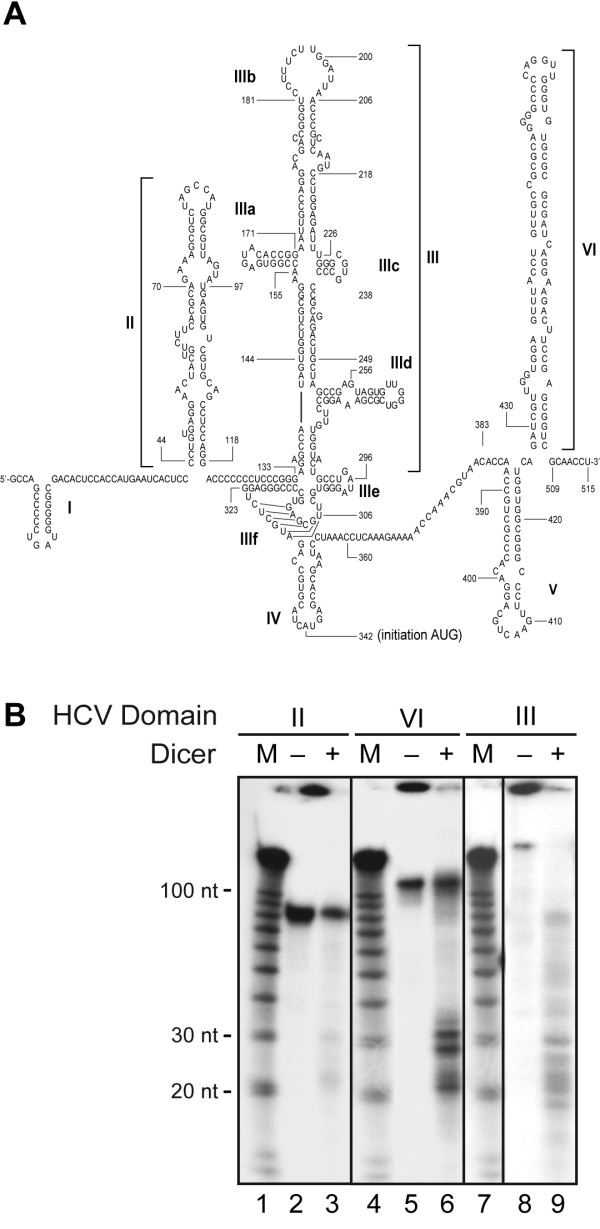
**HCV domains II, III and VI are processed into ~21 to 23-nt RNA species by recombinant human Dicer in vitro**. (A) Predicted secondary structure of nt 1 to 515 of the HCV RNA genome. (B) Dicer RNase activity assays. ^32^P-labeled HCV RNA domain II (left panel), domain VI (center panel) or domain III (right panel) was incubated in the absence (-) or presence (+) of recombinant human Dicer (65 ng) with MgCl_2_. The samples were analyzed by denaturing PAGE and autoradiography. M, indicates a 10-nt RNA size marker.

We tested this hypothesis and examined the susceptibility of the isolated domains of the HCV IRES to Dicer processing in vitro. Domains II and VI, in particular, show structural features of pre-miRNAs, such as a stem of imperfect complementarity long enough to be processed by a bidentate RNase III, the presence of a loop as well as of small bulges (see Fig. 3A). The HCV domain III structure, however, differs slightly from that of common pre-miRNAs, in that extended bulges forming distinct stem-loop entities, defined as domains IIIa, IIIc and IIId, are connected to the central stem (see Fig. 3A). We thus prepared ^32^P-labeled RNA substrates corresponding to HCV domain II (nt 42-120), domain III (nt 132 to 292) and domain VI (nt 426-510) by in vitro transcription and confirmed their ability to be recognized by recombinant human Dicer in EMSA experiments in vitro (I. Plante and P. Provost, unpublished data). Activation of the RNase III function of Dicer, upon addition of the divalent cation Mg^2+^, induced the processing of HCV domain II, III and VI RNAs into small, ~21 to 28 nt RNA species (see Fig. 3B, lanes 3, 6 and 9). The presence of small RNA species of ~22 nt derived from HCV domains II and III that suggest that these domains are less prone to Dicer cleavage when they are embedded within the HCV IRES nt1-341 RNA (compare with Fig. 2C, left panel). HCV IRES domain VI also appears to be more efficiently cleaved by Dicer as compared to domains II and III, which is in agreement with the observation that the HCV IRES nt1-515 cleavage is processed more efficiently than the HCV IRES nt 1-341 substrate (see Fig. 2C).

### Dicer does not bind HCV IRES in vivo

**Figure 4:**
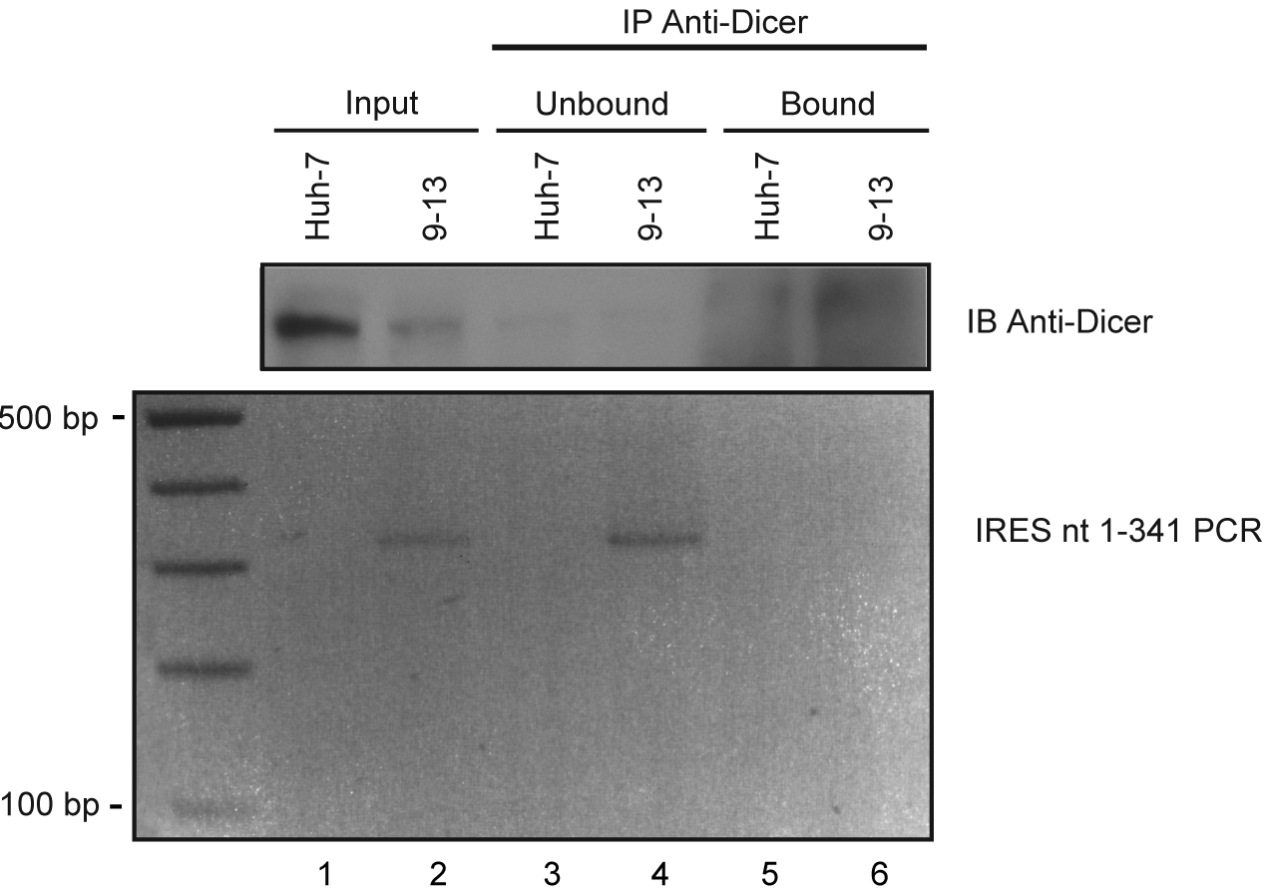
**Dicer does not bind HCV IRES in vivo**. HCV IRES nt 1-341 was amplified by RT-PCR from RNA extracted from Dicer immunoprecipitates (IPs) prepared from Huh-7 or 9–13 cells by ribonucleoprotein immunoprecipitation (RIP) assay. The amplified DNA products were analyzed by 1.5% agarose gel electrophoresis and stained with ethidium bromide (lower panel). Proteins (100 μg) were analyzed by 10% SDS-PAGE to visualize Dicer protein expression or immunoprecipitation in Huh-7 and 9–13 cells (upper panel).

These results led us to assess whether Dicer could bind the HCV IRES in vivo. We examined that issue by ribonucleoprotein immunoprecipitation (RIP) assay in 9–13 and Huh-7 cells, followed by reverse transcription (RT) and polymerase chain reaction (PCR) amplification of the HCV IRES from the immunoprecipitates (IPs). Western blot analyses revealed a large proportion of Dicer protein in input and IP (see Fig. 4, lanes 1, 2, 5 and 6), as expected. Unfortunately, we were unable to detect HCV IRES RNA in Dicer IPs (see Fig. 4, lower panel, lane 6), whereas the presence of the HCV IRES could be detected in the cell lysate (input) and the unbound fraction of the IP-Dicer prepared from 9–13 cells (see Fig. 4, upper panel, lanes 2 and 4).

Northern blot analyses and RNase protection assays (RPA), which have been found to be suitable for the detection of miRNAs derived from HIV-1 TAR RNA in vivo [29], did not allow the detection of small RNA species derived from the HCV IRES domain II or III (domain VI is absent from subgenomic HCV replicons) among a population of small RNAs (< 200 nt) extracted from 9–13 cells carrying the HCV replicon I_377_/NS3-3' from genotype 1b [47] (D.L. Ouellet and P. Provost, unpublished data). In HEK 293 cells, the level of small RNA species derived from a prototypic IRES-Rluc reporter mRNA, in the absence of HCV non-structural protein expression, also remained below the detection limit of our methods (D.L. Ouellet and P. Provost, unpublished data). Our inability to detect HCV IRES-derived small RNAs suggests that the HCV IRES may adopt a conformation that confers a certain degree of resistance to the recognition and processing activity of Dicer. It is also possible that the HCV IRES is not accessible to Dicer in a cellular context.

### Expression of Dicer does not alter HCV IRES-mediated translation

**Figure 5:**
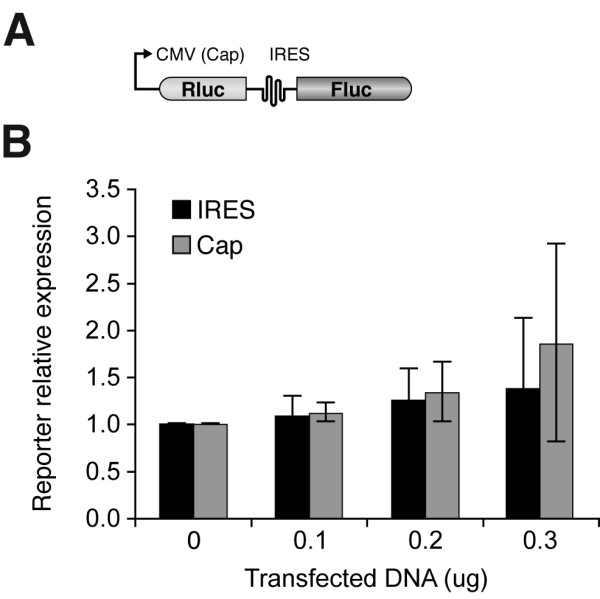
**Overexpression of Dicer has no effect on HCV IRES-mediated translation**. (A) Schematic representation of the reporter gene construct with pRL-CMV-1-515. (B) Reporter gene activity assays. pRL-CMV-1-515 was co-transfected in HEK 293 cells with increasing amounts (0–300 ng DNA) of pcDNA3.1-5'Flag-Dicer. Cells were harvested seventy-two (72) hours later, lysates were prepared, and Rluc and Fluc activities were measured successively. The results were normalized with those obtained from cells cotransfected with pRL-CMV-1-515 with empty vector pcDNA3.1-5'Flag. Results are expressed as mean ± s.e.m. (n = 6 experiments, in duplicate).

In light of these findings, we reexamined the relationship between Dicer and HCV domains II, III and VI in the context of the full-length IRES and, more specifically, assessed the influence of Dicer on the ability of the HCV IRES to mediate translation in vivo. To address that issue, we developed a bicistronic vector, called pRL-CMV-1-515, in which the Rluc reporter gene is under the control of the cap-dependent CMV promoter and the Fluc reporter gene driven by the HCV IRES nt 1-515 (see Fig. 5A). For these HCV IRES-mediated translation assays, HEK 293 cells were cotransfected with pRL-CMV-1-515 and increasing amounts of Dicer expression vector. As shown in Fig. 5B, Dicer overexpression had no effect on reporter gene expression driven by the HCV IRES. Similar conclusions were reached when using a bicistronic vector (pRL-CMV-I371) in which nt 1-371 of the HCV IRES are placed upstream of the Fluc reporter (D.L. Ouellet and P. Provost, unpublished data), suggesting that Dicer overexpression does not alter HCV IRES-mediated translation in vivo.

## Discussion

The interplay between viruses and the RNA silencing machinery of the hosts is increasingly complex, as reviewed recently for HIV-1 [48]. Some viruses, such as HIV-1 [49] and adenoviruses [44], have efficiently adapted to small RNA-based host defense mechanisms and evolved inhibitors of Dicer function.

In the case of HCV, we observed that expression of its non-structural proteins from a subgenomic replicon had no effect on the efficiency of RNA silencing induced by a pre-miRNA or sh RNA Dicer substrate, or downstream of it (D. Ouellet, I. Plante, and P. Provost, unpublished data). This is in accordance with a previous study by Kanda et al [41], which has demonstrated the efficacy of a shRNA directed against HCV to inhibit viral replication in replicon-containing Huh-7 cells. However, it has been reported more recently that the HCV structural proteins core and E2, which are not part of our subgenomic replicon model, could interact with Dicer and Ago2, respectively [34-36]. Indeed, it was shown that the HCV core protein may abrogate RNA silencing induced by shRNAs, but not that induced by siRNAs, in HepG2 hepatocytes and non-hepatocyte mammalian cells expressing only the HCV core [34]. The decreased efficiency of a shRNA directed against HCV RNA in cells carrying a genomic versus a subgenomic replicon, as observed by Kanda et al. [41], may thus be related to a Dicer inhibitory effect of the HCV core protein [41]. A recent paper also showed that the HCV E2 envelope protein interacts with Ago2, the catalytic engine of the RNA-induced silencing complex (RISC), suggesting that HCV proteins may inhibit RNA silencing pathways at different steps.

These observations, however, are in contrast to a previous report showing, that the endogenous level of three different miRNAs remained unchanged in Huh-7 cells carrying an HCV genomic replicon [26]. These data militate against a role for the HCV core and E2 proteins as suppressors of RNA silencing, although monitoring the accumulation of the miRNA end-product may not always accurately reflect or be sensitive enough to detect slight alterations in the functionality of the whole miRNA-guided RNA silencing pathway. Considering that cellular miRNAs, such as miR-199a [50], could target the HCV genome and inhibit viral replication and that interferon could modulate expression of certain miRNAs that may either target the HCV RNA genome (eg, as miR-196 or miR-448) [51] or markedly enhance its replication (eg, miR-122) [42], it will be important to determine whether the HCV core and E2 proteins interferes with the host RNA silencing processes during the natural course of an HCV infection.

Some viruses, such as EBV [24], KSHV, HCMV [25,26] and HIV-1 [27-29], appear to be vulnerable to Dicer processing and thus represent a source of miRNAs that can potentially interfere with the gene expression programming of the host. We recently reported the ability of Dicer to release functional miRNAs from the HIV-1 TAR element [29], a stem-bulge-loop RNA located at the 5' extremity of all HIV-1 mRNAs transcripts. Employing the same strategy and experimental approaches [29], we were able to document the ability of human Dicer to cleave HCV IRES nt 1-341 and nt 1-515 RNAs as well as domains II, III and VI derived from the HCV IRES in vitro. Processing of the HCV IRES RNA by recombinant Dicer in vitro had been reported previously [35]. The pattern of the RNA products that we observed upon Dicer cleavage of either HCV IRES or that of its structural domains is compatible with imperfect substrate recognition by Dicer and/or an improper alignment of its RNase III domains at the expected cleavage sites that may result in asymmetrical processing of the HCV RNA substrate and yield RNA intermediate species. Mechanistically, endogenous substrate recognition by Dicer has been proposed to involve anchoring of the pre-miRNA 2-nt 3'overhang in the pocket formed by its central PAZ domain [52,53]. Devoid of defined 3'overhang, the HCV IRES is not a common substrate for Dicer. Imperfect HCV IRES recognition and processing by Dicer may thus explain, at least in part, the length heterogeneity of the resulting RNA products.

We were unable to document the presence of HCV IRES RNA in Dicer IP prepared from 9–13 cells by RIP assay, suggesting a lack of interaction between Dicer and the HCV IRES in vivo. Moreover, we could not detect small RNAs derived from the HCV IRES either by Northern Blot or RPA analyses. Although we cannot exclude the possibility that HCV miRNA levels remained below the sensitivity limit of our technique, our findings do not support the concept of HCV IRES binding and cleavage by Dicer in vivo. Although HCV is an RNA virus whose replication occurs in the endoplasmic reticulum and cytoplasmic compartments [1], the HCV IRES RNA and domains II, III and VI may not represent ideal Dicer substrates, as they are embedded within the HCV RNA genome. Recently, the relatively low processing reactivity of the HIV-1 TAR RNA to Dicer has been attributed, at least in part, to the lack of a free 3' end and its embedding at the 5' end of HIV-1 mRNAs [29]. The situation of HCV domains II, III and VI may also be different from that reported for the *env *[27] and *nef *[28] regions of HIV-1, whose internal hairpin-loop precursor sequences may be located in a different, more favorable structural context. The unavailability of free 5' and 3' ends at the base of domains II, III and VI may thus account, at least in part, for the relative refractoriness of the HCV IRES to processing by Dicer.

A limited accessibility to the viral RNA may also be a contributing factor to the relative lack of reactivity of HCV IRES to Dicer in vivo. In support to this hypothesis is the lack of effects of Dicer overexpression on the HCV IRES-mediated translation in HEK 293 cells (D.L. Ouellet and P. Provost, unpublished data), which are devoid of HCV non-structural proteins suggesting that the HCV IRES remains inaccessible to Dicer even in the absence of HCV proteins. However, this possibility has been challenged by a recent study showing that miR-122 modulates HCV RNA abundance in Huh-7 cell stably expressing the genotype 1b strain HCV-N replicon NNeo/C-5B [42]. MiR-122 has been proposed to act through recognition of two putative binding sites, one of which is located in the HCV 5'UTR upstream of domain II. In that context, the observed miRNA regulation, which is usually mediated by the RISC effector complex, imply a certain degree of accessibility to specific sequences within the HCV IRES. This interpretation is further supported by the efficiency of an shRNA directed against domain II of HCV IRES at reducing the level of HCV 5'NTR RNA in Huh-7 cells carrying a genomic replicon [41]. On the other hand, no miRNAs derived from the virus could be detected among 1318 small RNA sequences isolated from the Huh-7.5 cell line [26]. These observations suggest a differential access of a miR-122/RISC complex, versus that of a pre-miRNA processing complex containing Dicer, to the IRES structure of HCV in vivo. It could be hypothesized that the Dicer protein has no access to the HCV IRES RNA despite its possible presence within RISC complexes [54,55], and that access is somehow restricted to other proteins of the RISC complex, such as Ago2. Moreover, since HCV-derived miRNAs may be expressed at very low levels, among an abundant amount of cellular miRNAs, they could have escaped detection by standard small RNA cloning strategies, as we previously reported for miR-TAR-3p and miR-TAR-5p released from HIV-1 TAR RNA [29].

Viral and cellular proteins interacting with the HCV IRES, in the context of viral replication and/or mRNA translation, are likely to further decrease the vulnerability of these structures to Dicer processing in vivo. Among these factors are the polypyrimidine-tract-binding protein [56], the human La antigen [56,57], the poly(rC)-binding protein 2 [58], the heterogeneous nuclear ribonucleoprotein L [59], proteasome α-subunit PSMA7 [60] and probably many others [61]. In support to this assertion, siRNA-mediated suppression of Hu antigen R (HuR) and PSMA7 substantially diminished HCV IRES-mediated translation and subgenomic HCV replication [62]. In addition, suppression of La antigen expression with antisense phosphorothioate oligonucleotides reduced HCV IRES activity from a bicistronic vector [63]. The possibility that these IRES-interacting proteins can shield this key viral RNA structure from the processing activity of Dicer is attractive and warrant further investigations.

## Conclusion

HCV and the host RNA silencing machineries are likely engaged in a host-pathogen "arms race" that may be constantly shaping the virus genome as well as the antiviral functionalities of the host defense system. Our study suggests that the HCV IRES may have evolved to adopt a structure efficient in translation initiation and permissive to miR-122-mediated facilitation of viral replication, while exhibiting refractoriness to processing by Dicer. These properties of the HCV IRES, which may be governed by sequestration of HCV RNA in the replication complex as well as by various interactions with viral and cellular proteins, may contribute to viral escape of the host RNA silencing machinery and persistence in infected individuals.

## Methods

### Mammalian cell culture

Huh-7 and 9–13 cells were maintained in DMEM supplemented with 10% fetal bovine serum, 1× non-essential amino acids, 2 mM L-glutamine, 100 units/ml penicillin and 100 μg/ml streptomycin in a humidified incubator under 5% CO_2 _at 37°C. HCV replicon I_377_/NS3-3'-containing 9–13 cells were kept under selection with 1 μg/ml of G418. Cured cells were generated upon treatment with 100 IU/ml of IFNα-2B (Intron^® ^A, Schering) for 4 to 6 passages, as described previously [45,46]. HEK 293 cells were grown in DMEM supplemented with 10% fetal bovine serum, 1 mM sodium pyruvate, 2 mM L-glutamine, 100 units/ml penicillin and 100 μg/ml streptomycin in a humidified incubator under 5% CO_2 _at 37°C.

### Western and Northern blot analyses

Dicer, HCV NS3, NS5B and actin proteins were detected by Western blot using rabbit anti-Dicer [18], mouse anti-NS3 IB6 [64], anti-NS5B 5B-3B1 [65] and anti-actin AC-40 (Sigma) antibodies, respectively. HCV IRES RNA was detected by Northern blotting using a DNA probe complementary to HCV nt 1-341, whereas a DNA probe recognizing GAPDH mRNA was used as a loading control.

### MicroRNA-guided RNA silencing activity assay

The pre-miR-328 expression vector was conceived by cloning in psiSTRIKE the pre-mmu-miR-328 sequence (5'accgtggagtgggggggcaggaggggctcagggagaaagtgcatacagcccctggccctctctgcccttccgtcccctgt ttttc-3') (Promega). The Rluc:miR-328 binding site reporter constructs, in which Rluc is coupled with 1 or 3 copies of perfectly complementary (PC) or natural wild-type (WT) binding sites for mmu-miR-328, were obtained by cloning 1 or 3 copies of the PC (5'-atctcaacggaagggcagagagggccagatctc-3') or WT (5'-atctcgtccctgtggtaccctggcagagaaagggccaatctcaatctc-3') binding sites into the PmeI site of psiCHECK (Promega). The integrity of the constructs was verified by restriction analysis and DNA sequencing (CHUQ Research Center DNA sequencing core facility).

To estimate the efficiency of RNA silencing, Huh-7 and 9–13 cells were grown in 24-well plates to reach ~70% confluency prior to transfection using Lipofectamine 2000 (Invitrogen) with either psiCHECK (0.4 μg DNA) and psiRluc or psiNeg (0.25–250 ng DNA), or Rluc:miR-328 BS reporter constructs (0.4 ng DNA) and pre-mmu-miR-328 expression construct (250 ng DNA). Cells were harvested 24 hours later, lysates were prepared, and luciferase activities were measured, as described previously [66].

### Dicer RNase activity assay

The HCV IRES domains II, III, and VI, as well as HCV IRES RNAs were transcribed and randomly labeled (α-^32^P UTP, Perkin Elmer) by in vitro transcription using T7 promoter (MEGAshort Script kit, Ambion), and purified by denaturating PAGE (5%). ^32^P-labeled HCV RNAs (30 000 cpm) were incubated in the absence or presence of recombinant human Dicer (65 ng prot) with MgCl_2 _(5 mM) at 37°C for 1 h. The reaction was analyzed by denaturing PAGE (10%) and the resulting RNA products were detected by autoradiography, as described previously [18,66].

### Electrophoretic mobility shift assay (EMSA)

The HCV IRES nt 1-515 and 1-341 RNAs were transcribed and randomly labeled (α-^32^P UTP, Perkin Elmer) by in vitro transcription using T7 promoter (MEGAshort Script kit, Ambion), and purified by denaturating PAGE (5%). ^32^P-labeled HCV IRES RNAs (30 000 cpm) were incubated in the absence or presence of recombinant human Dicer (200 ng prot) [18], with or without BSA (2 μg), for 30 min on ice prior to electrophoretic mobility shift assay (EMSA) analysis, which was performed as described previously [18,66]. Dicer•HCV IRES RNA complex formation was analyzed by nondenaturating PAGE (6%) and detected by autoradiography.

### Ribonucleoprotein immunoprecipitation (RIP) assay

Huh-7 and 9–13 cells were grown to reach ~70% confluency in 10-cm culture dishes and harvested in 10 ml of PBS 1×, as described previously [67]. Briefly, cells were fixed with formaldehyde (37% in 10% methanol) to a final concentration of 1% (v/v, 0.36 M) and incubated at room temperature for 10 minutes with slow mixing. The crosslinking reaction was quenched upon addition of glycine (pH 7.0) to a final concentration of 0.25 M and incubation at room temperature for 5 minutes. Cells were harvested by centrifugation at 237 *g *for 4 minutes, followed by two washes with ice-cold PBS. The pellet was resuspended in 1 ml of RIPA buffer (Tris·HCl 50 mM, NP-40 1%, Sodium deoxycholate 0.5%, EDTA 1 mM, Sodium dodecyl sulphate 0.05% and 150 mM NaCl, pH 7.5) and the protein·RNA species crosslinked were solubilised by sonication. After removal of the insoluble material by centrifugation at 16 000 *g *for 10 minutes, the supernatant was precleared with protein G agarose and non-specific tRNA competitor at a final concentration of 100 μg/ml. After incubating for 1 h at 4°C, the sample was centrifuged and an aliquot was kept for RNA extraction (input) and Western blot analysis. The precleared lysate was further incubated with precomplexed protein G/rabbit anti-Dicer for 90 minutes at 4°C with rotation for immunoprecipitation of the crosslinked Dicer·RNA species. The beads were collected by centrifugation at 600 *g *for 1 minute, washed 5 times with RIPA High Stringency buffer (Tris·HCl 50 mM, NP-40 1%, Sodium deoxycholate 1%, EDTA 1 mM, Sodium dodecyl sulphate 0.1%, 1 M NaCl, 1 M Urea, pH 7.5) and resuspended in 100 μl of resuspension buffer (Tris·HCl 50 mM, EDTA 5 mM, DTT 10 mM and Sodium dodecyl sulphate 1%, pH 7.0), as described previously [67]. An aliquot of the first supernatant (unbound fraction) was kept for RNA extraction and Western blot analysis. The beads were then was incubated 45 minutes at 70°C to reverse the crosslinks and RNA was extracted with TRIZOL reagent.

The RNA was subjected to RT using specific primer to the neomycin region of the HCV RNA (5'-TGGCCAGCCACGATAGCCGC-3') with SuperScript II (Invitrogen), according to the manufacturer's instructions. The polymerase chain reaction (PCR) was performed using the Phusion polymerase (NEB) and the HCV nt 1-341 fragment was amplified with forward (5'-gattgggggcgacactccac-3') and reverse (5'-tacgagacctcccggggcac-3') oligonucleotides.

### HCV IRES-mediated translation assay

The HCV IRES nt 1-515 segment was amplified by PCR from pHCV77c using forward (5'-gcgcgcggatccgccagccccctgatgggggcgacac-3') and reverse (5'-gcgcgcggatccaggttgcgaccgctcggaagtcttcc-3') oligonucleotides, and cloned in the BamHI site of pXP2-Luc (*Firefly *luciferase) vector. The IRES 1-515/Fluc unit was then reamplified by PCR using forward (5'-gcgcgcactagtgccagccccctgatgggggcgacac-3') and reverse (5'-gcgcgcactagtttacaatttggactttccgcccttc-3') oligonucleotides, and transferred to the XbaI/BamHI sites of pRL-CMV vector (Promega).

In order to document the effects of Dicer overexpression on HCV IRES function, HEK 293 cells grown in 24-well plates to ~50% confluency were cotransfected with pRL-CMV-1-515 (100 ng DNA) and pcDNA3.1-5'Flag-Dicer (0–300 ng DNA) [18], or pcDNA3.1 empty vector (0–300 ng DNA). Cells were harvested 72 hours later, lysates were prepared, and Rluc and Fluc activities were measured successively using the Dual-Luciferase Reporter Assay System (Promega), as described previously [29].

## Competing interests

The authors declare that they have no competing interests.

## Authors' contributions

DLO, IP and CA performed the experiments and analyzed the data. VB developed a new research tool. PP conceived the study. DLO and PP wrote the manuscript. All authors read and approved the final manuscript.

## Supplementary Material

Additional file 1 **VA1 RNA from adenovirus interfere with RNA silencing in Huh-7 cells. The data provided attest of the suitability of our reporter gene system to assess the influence of HCV non-structural proteins on the host miRNA-guided RNA silencing machinery**. Huh-7 cells were cotransfected using Lipofectamine 2000 with psiCHECK (400 ng DNA), psiSTRIKE (Rluc or Neg, 250 ng DNA) and increasing amount of pBS II KS(+) (pBS) or pBS II KS(+) VA1 (pBS VA1) vectors (10–400 ng DNA). The pBS VA1 expression vector was prepared through amplification of a 330-nt VA1 fragment, containing sequences for RNA polymerase III transcription, from pADEasy vector (Stratagene) by using forward (5'-gagagagaattccggtcgggacgctctggcc-3') and reverse (5'gcgcgcaagcttcttaatgctttcgctttcc-3') oligonucleotides, and cloned in the EcoRI/HindIII sites of pBluescript II KS(+) vector (Invitrogen), as described in Lu and Cullen [44]. psiSTRIKE-Neg was used as a control. Results of Rluc activity were normalized with Fluc activity, and expressed as a percentage of Rluc activity obtained with a shRNA (Neg) directed against a sequence deleted in the Rluc reporter mRNA. Results are expressed as mean ± s.e.m. (n = 2 to 3 experiments, in duplicate). Click here for file

Additional file 2 **Dicer in functionally competent in Huh-7 and 9–13 cells. The data provided indicate that the activity of Dicer is not influenced by HCV in vivo**. The human pre-let7a-3 RNA was transcribed and randomly labeled (α-^32^P UTP, Perkin Elmer) by in vitro transcription using T7 promoter (Ambion) and purified by 10% denaturating PAGE. Huh-7 and 9–13 cells were resuspended in lysis buffer (Tris·HCl 50 mM, 137 mM NaCl, Triton X-100 1%) and immunoprecipitation (IP) was performed on 1 mg of proteins incubated with protein-G beads alone or beads/rabbit anti-Dicer at 4°C for 3 hours. Immune complexes were washed 3 times in lysis buffer, following by an additional wash in Tris·HCl 20 mM and MgCl_2 _2 mM, pH 7.5. α-^32^P labeled pre-let7a-3 RNA was incubated with immune complexes for in vitro processing of pre-miRNA in Dicer RNase activity assay for 1 hour at 37°C in Tris·HCl 20 mM, DTT 1 mM, ATP 1 mM, MgCl_2 _5 mM and 5% SUPERase∙In (Ambion), pH 7.5. Proteins were extracted by phenol/chloroform and RNA was precipitated and analyzed by denaturating PAGE and autoradiography. Click here for file

## References

[B1] Reed KE, Rice CM (2000). Overview of hepatitis C virus genome structure, polyprotein processing, and protein properties. Curr Top Microbiol Immunol.

[B2] McHutchison JG, Patel K (2002). Future therapy of hepatitis C. Hepatology.

[B3] Choo QL, Richman KH, Han JH, Berger K, Lee C, Dong C, Gallegos C, Coit D, Medina-Selby R, Barr PJ (1991). Genetic organization and diversity of the hepatitis C virus. Proc Natl Acad Sci USA.

[B4] Grakoui A, Wychowski C, Lin C, Feinstone SM, Rice CM (1993). Expression and identification of hepatitis C virus polyprotein cleavage products. J Virol.

[B5] Hellen CU, Pestova TV (1999). Translation of hepatitis C virus RNA. J Viral Hepat.

[B6] Rijnbrand RC, Lemon SM (2000). Internal ribosome entry site-mediated translation in hepatitis C virus replication. Curr Top Microbiol Immunol.

[B7] Tsukiyama-Kohara K, Iizuka N, Kohara M, Nomoto A (1992). Internal ribosome entry site within hepatitis C virus RNA. J Virol.

[B8] Wang C, Sarnow P, Siddiqui A (1993). Translation of human hepatitis C virus RNA in cultured cells is mediated by an internal ribosome-binding mechanism. J Virol.

[B9] Robert F, Kapp LD, Khan SN, Acker MG, Kolitz S, Kazemi S, Kaufman RJ, Merrick WC, Koromilas AE, Lorsch JR (2006). Initiation of Protein Synthesis by Hepatitis C Virus Is Refractory to Reduced eIF2*GTP*Met-tRNAiMet Ternary Complex Availability. Mol Biol Cell.

[B10] Boni S, Lavergne JP, Boulant S, Cahour A (2005). Hepatitis C virus core protein acts as a trans-modulating factor on internal translation initiation of the viral RNA. J Biol Chem.

[B11] He Y, Yan W, Coito C, Li Y, Gale M, Katze MG (2003). The regulation of hepatitis C virus (HCV) internal ribosome-entry site-mediated translation by HCV replicons and nonstructural proteins. J Gen Virol.

[B12] Kato J, Kato N, Yoshida H, Ono-Nita SK, Shiratori Y, Omata M (2002). Hepatitis C virus NS4A and NS4B proteins suppress translation in vivo. J Med Virol.

[B13] Kou YH, Chou SM, Wang YM, Chang YT, Huang SY, Jung MY, Huang YH, Chen MR, Chang MF, Chang SC (2006). Hepatitis C virus NS4A inhibits cap-dependent and the viral IRES-mediated translation through interacting with eukaryotic elongation factor 1A. J Biomed Sci.

[B14] Shimoike T, Koyama C, Murakami K, Suzuki R, Matsuura Y, Miyamura T, Suzuki T (2006). Down-regulation of the internal ribosome entry site (IRES)-mediated translation of the hepatitis C virus: critical role of binding of the stem-loop IIId domain of IRES and the viral core protein. Virology.

[B15] Honda M, Beard MR, Ping LH, Lemon SM (1999). A phylogenetically conserved stem-loop structure at the 5' border of the internal ribosome entry site of hepatitis C virus is required for cap-independent viral translation. J Virol.

[B16] Lukavsky PJ, Otto GA, Lancaster AM, Sarnow P, Puglisi JD (2000). Structures of two RNA domains essential for hepatitis C virus internal ribosome entry site function. Nat Struct Biol.

[B17] Lee Y, Ahn C, Han J, Choi H, Kim J, Yim J, Lee J, Provost P, Radmark O, Kim S (2003). The nuclear RNase III Drosha initiates microRNA processing. Nature.

[B18] Provost P, Dishart D, Doucet J, Frendewey D, Samuelsson B, Radmark O (2002). Ribonuclease activity and RNA binding of recombinant human Dicer. Embo J.

[B19] Zhang H, Kolb FA, Brondani V, Billy E, Filipowicz W (2002). Human Dicer preferentially cleaves dsRNAs at their termini without a requirement for ATP. Embo J.

[B20] Bartel DP (2004). MicroRNAs: genomics, biogenesis, mechanism, and function. Cell.

[B21] Ouellet DL, Perron MP, Gobeil LA, Plante P, Provost P (2006). MicroRNAs in Gene Regulation: When the Smallest Governs It All. J Biomed Biotechnol.

[B22] Hamilton AJ, Baulcombe DC (1999). A species of small antisense RNA in posttranscriptional gene silencing in plants. Science.

[B23] Reinhart BJ, Weinstein EG, Rhoades MW, Bartel B, Bartel DP (2002). MicroRNAs in plants. Genes Dev.

[B24] Pfeffer S, Zavolan M, Grasser FA, Chien M, Russo JJ, Ju J, John B, Enright AJ, Marks D, Sander C (2004). Identification of virus-encoded microRNAs. Science.

[B25] Cai X, Lu S, Zhang Z, Gonzalez CM, Damania B, Cullen BR (2005). Kaposi's sarcoma-associated herpesvirus expresses an array of viral microRNAs in latently infected cells. Proc Natl Acad Sci USA.

[B26] Pfeffer S, Sewer A, Lagos-Quintana M, Sheridan R, Sander C, Grasser FA, van Dyk LF, Ho CK, Shuman S, Chien M (2005). Identification of microRNAs of the herpesvirus family. Nat Methods.

[B27] Bennasser Y, Le SY, Benkirane M, Jeang KT (2005). Evidence that HIV-1 encodes an siRNA and a suppressor of RNA silencing. Immunity.

[B28] Omoto S, Ito M, Tsutsumi Y, Ichikawa Y, Okuyama H, Brisibe EA, Saksena NK, Fujii YR (2004). HIV-1 nef suppression by virally encoded microRNA. Retrovirology.

[B29] Ouellet DL, Plante I, Landry P, Barat C, Janelle ME, Flamand L, Tremblay MJ, Provost P (2008). Identification of functional microRNAs released through asymmetrical processing of HIV-1 TAR element. Nucleic Acids Res.

[B30] Merai Z, Kerenyi Z, Kertesz S, Magna M, Lakatos L, Silhavy D (2006). Double-stranded RNA binding may be a general plant RNA viral strategy to suppress RNA silencing. J Virol.

[B31] Li WX, Li H, Lu R, Li F, Dus M, Atkinson P, Brydon EW, Johnson KL, Garcia-Sastre A, Ball LA (2004). Interferon antagonist proteins of influenza and vaccinia viruses are suppressors of RNA silencing. Proc Natl Acad Sci USA.

[B32] Li H, Li WX, Ding SW (2002). Induction and suppression of RNA silencing by an animal virus. Science.

[B33] Soldan SS, Plassmeyer ML, Matukonis MK, Gonzalez-Scarano F (2005). La Crosse virus nonstructural protein NSs counteracts the effects of short interfering RNA. J Virol.

[B34] Chen W, Zhang Z, Chen J, Zhang J, Wu Y, Huang Y, Cai X, Huang A (2008). HCV core protein interacts with Dicer to antagonize RNA silencing. Virus Res.

[B35] Wang Y, Kato N, Jazag A, Dharel N, Otsuka M, Taniguchi H, Kawabe T, Omata M (2006). Hepatitis C virus core protein is a potent inhibitor of RNA silencing-based antiviral response. Gastroenterology.

[B36] Ji J, Glaser A, Wernli M, Berke JM, Moradpour D, Erb P (2008). Suppression of short interfering RNA-mediated gene silencing by the structural proteins of hepatitis C virus. J Gen Virol.

[B37] Randall G, Grakoui A, Rice CM (2003). Clearance of replicating hepatitis C virus replicon RNAs in cell culture by small interfering RNAs. Proc Natl Acad Sci USA.

[B38] Wilson JA, Jayasena S, Khvorova A, Sabatinos S, Rodrigue-Gervais IG, Arya S, Sarangi F, Harris-Brandts M, Beaulieu S, Richardson CD (2003). RNA interference blocks gene expression and RNA synthesis from hepatitis C replicons propagated in human liver cells. Proc Natl Acad Sci USA.

[B39] Yokota T, Sakamoto N, Enomoto N, Tanabe Y, Miyagishi M, Maekawa S, Yi L, Kurosaki M, Taira K, Watanabe M (2003). Inhibition of intracellular hepatitis C virus replication by synthetic and vector-derived small interfering RNAs. EMBO Rep.

[B40] Hamazaki H, Takahashi H, Shimotohno K, Miyano-Kurosaki N, Takaku H (2006). Inhibition of hcv replication in HCV replicon by shRNAs. Nucleosides Nucleotides Nucleic Acids.

[B41] Kanda T, Steele R, Ray R, Ray RB (2007). Small interfering RNA targeted to hepatitis C virus 5' nontranslated region exerts potent antiviral effect. J Virol.

[B42] Jopling CL, Yi M, Lancaster AM, Lemon SM, Sarnow P (2005). Modulation of hepatitis C virus RNA abundance by a liver-specific MicroRNA. Science.

[B43] Boissonneault V, Plante I, Rivest S, Provost P (2009). MicroRNA-298 and MicroRNA-328 Regulate Expression of Mouse {beta}-Amyloid Precursor Protein-converting Enzyme 1. J Biol Chem.

[B44] Lu S, Cullen BR (2004). Adenovirus VA1 noncoding RNA can inhibit small interfering RNA and MicroRNA biogenesis. J Virol.

[B45] Frese M, Pietschmann T, Moradpour D, Haller O, Bartenschlager R (2001). Interferon-alpha inhibits hepatitis C virus subgenomic RNA replication by an MxA-independent pathway. J Gen Virol.

[B46] Prabhu R, Joshi V, Garry RF, Bastian F, Haque S, Regenstein F, Thung S, Dash S (2004). Interferon alpha-2b inhibits negative-strand RNA and protein expression from full-length HCV1a infectious clone. Exp Mol Pathol.

[B47] Lohmann V, Korner F, Koch J, Herian U, Theilmann L, Bartenschlager R (1999). Replication of subgenomic hepatitis C virus RNAs in a hepatoma cell line. Science.

[B48] Provost P, Barat C, Plante I, Tremblay MJ (2006). HIV-l and the microRNA-guided silencing pathway: An intricate and multifaceted encounter. Virus Res.

[B49] Bennasser Y, Jeang KT (2006). HIV-1 Tat interaction with Dicer: requirement for RNA. Retrovirology.

[B50] Murakami Y, Aly HH, Tajima A, Inoue I, Shimotohno K (2008). Regulation of the hepatitis C virus genome replication by miR-199a. J Hepatol.

[B51] Pedersen IM, Cheng G, Wieland S, Volinia S, Croce CM, Chisari FV, David M (2007). Interferon modulation of cellular microRNAs as an antiviral mechanism. Nature.

[B52] Ma JB, Ye K, Patel DJ (2004). Structural basis for overhang-specific small interfering RNA recognition by the PAZ domain. Nature.

[B53] Zhang H, Kolb FA, Jaskiewicz L, Westhof E, Filipowicz W (2004). Single processing center models for human Dicer and bacterial RNase III. Cell.

[B54] Chendrimada TP, Gregory RI, Kumaraswamy E, Norman J, Cooch N, Nishikura K, Shiekhattar R (2005). TRBP recruits the Dicer complex to Ago2 for microRNA processing and gene silencing. Nature.

[B55] Maniataki E, Mourelatos Z (2005). A human, ATP-independent, RISC assembly machine fueled by pre-miRNA. Genes Dev.

[B56] Ali N, Siddiqui A (1995). Interaction of polypyrimidine tract-binding protein with the 5' noncoding region of the hepatitis C virus RNA genome and its functional requirement in internal initiation of translation. J Virol.

[B57] Pudi R, Abhiman S, Srinivasan N, Das S (2003). Hepatitis C virus internal ribosome entry site-mediated translation is stimulated by specific interaction of independent regions of human La autoantigen. J Biol Chem.

[B58] Fukushi S, Okada M, Kageyama T, Hoshino FB, Nagai K, Katayama K (2001). Interaction of poly(rC)-binding protein 2 with the 5'-terminal stem loop of the hepatitis C-virus genome. Virus Res.

[B59] Hahm B, Kim YK, Kim JH, Kim TY, Jang SK (1998). Heterogeneous nuclear ribonucleoprotein L interacts with the 3' border of the internal ribosomal entry site of hepatitis C virus. J Virol.

[B60] Kruger M, Beger C, Welch PJ, Barber JR, Manns MP, Wong-Staal F (2001). Involvement of proteasome alpha-subunit PSMA7 in hepatitis C virus internal ribosome entry site-mediated translation. Mol Cell Biol.

[B61] Lu H, Li W, Noble WS, Payan D, Anderson DC (2004). Riboproteomics of the hepatitis C virus internal ribosomal entry site. J Proteome Res.

[B62] Korf M, Jarczak D, Beger C, Manns MP, Kruger M (2005). Inhibition of hepatitis C virus translation and subgenomic replication by siRNAs directed against highly conserved HCV sequence and cellular HCV cofactors. J Hepatol.

[B63] Honda M, Shimazaki T, Kaneko S (2005). La protein is a potent regulator of replication of hepatitis C virus in patients with chronic hepatitis C through internal ribosomal entry site-directed translation. Gastroenterology.

[B64] Wolk B, Sansonno D, Krausslich HG, Dammacco F, Rice CM, Blum HE, Moradpour D (2000). Subcellular localization, stability, and trans-cleavage competence of the hepatitis C virus NS3-NS4A complex expressed in tetracycline-regulated cell lines. J Virol.

[B65] Moradpour D, Bieck E, Hugle T, Wels W, Wu JZ, Hong Z, Blum HE, Bartenschlager R (2002). Functional properties of a monoclonal antibody inhibiting the hepatitis C virus RNA-dependent RNA polymerase. J Biol Chem.

[B66] Plante I, Davidovic L, Ouellet DL, Gobeil LA, Tremblay S, Khandjian EW, Provost P (2006). Dicer-Derived MicroRNAs Are Utilized by the Fragile X Mental Retardation Protein for Assembly on Target RNAs. J Biomed Biotechnol.

[B67] Niranjanakumari S, Lasda E, Brazas R, Garcia-Blanco MA (2002). Reversible cross-linking combined with immunoprecipitation to study RNA-protein interactions in vivo. Methods.

