# Supplementary figures and images for: Refractoriness of hepatitis C virus internal ribosome entry site to processing by Dicer in vivo
Source: J Negat Results Biomed. 2009 Aug 13;8:8. doi: 10.1186/1477-5751-8-8 (PMC2746800; doi:10.1186/1477-5751-8-8)

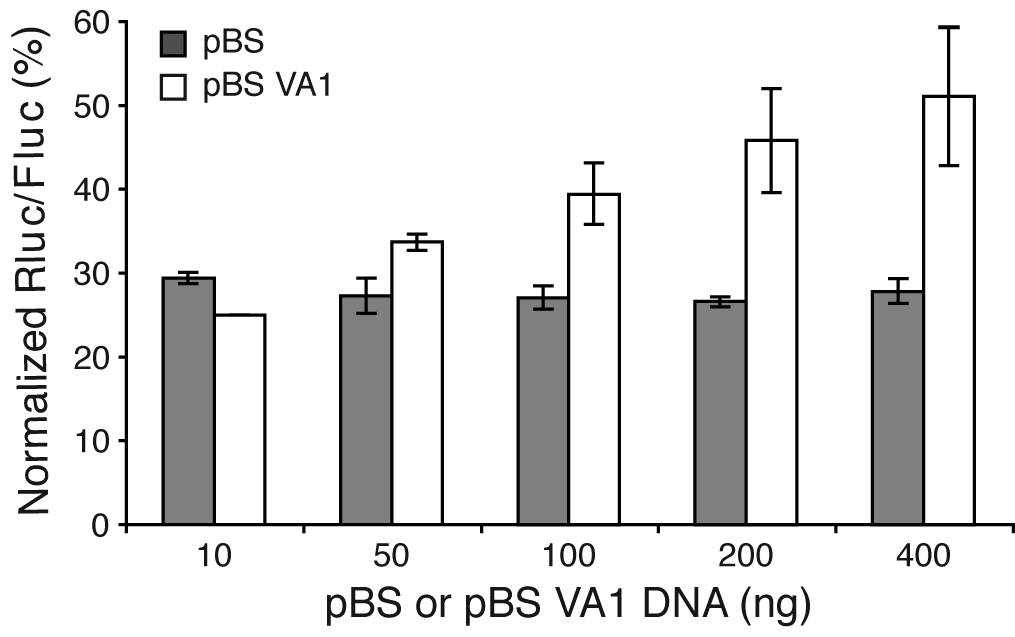

Supplement: Additional file 1 — VA1 RNA from adenovirus interfere with RNA silencing in Huh-7 cells. The data provided attest of the suitability of our reporter gene system to assess the influence of HCV non-structural proteins on the host miRNA-guided RNA silencing machinery. Huh-7 cells were cotransfected using Lipofectamine 2000 with psiCHECK (400 ng DNA), psiSTRIKE (Rluc or Neg, 250 ng DNA) and increasing amount of pBS II KS(+) (pBS) or pBS II KS(+) VA1 (pBS VA1) vectors (10–400 ng DNA). The pBS VA1 expression vector was prepared through amplification of a 330-nt VA1 fragment, containing sequences for RNA polymerase III transcription, from pADEasy vector (Stratagene) by using forward (5'-gagagagaattccggtcgggacgctctggcc-3') and reverse (5'gcgcgcaagcttcttaatgctttcgctttcc-3') oligonucleotides, and cloned in the EcoRI/HindIII sites of pBluescript II KS(+) vector (Invitrogen), as described in Lu and Cullen [44]. psiSTRIKE-Neg was used as a control. Results of Rluc activity were normalized with Fluc activity, and expressed as a percentage of Rluc activity obtained with a shRNA (Neg) directed against a sequence deleted in the Rluc reporter mRNA. Results are expressed as mean ± s.e.m. (n = 2 to 3 experiments, in duplicate). [file 1477-5751-8-8-S1.tiff]

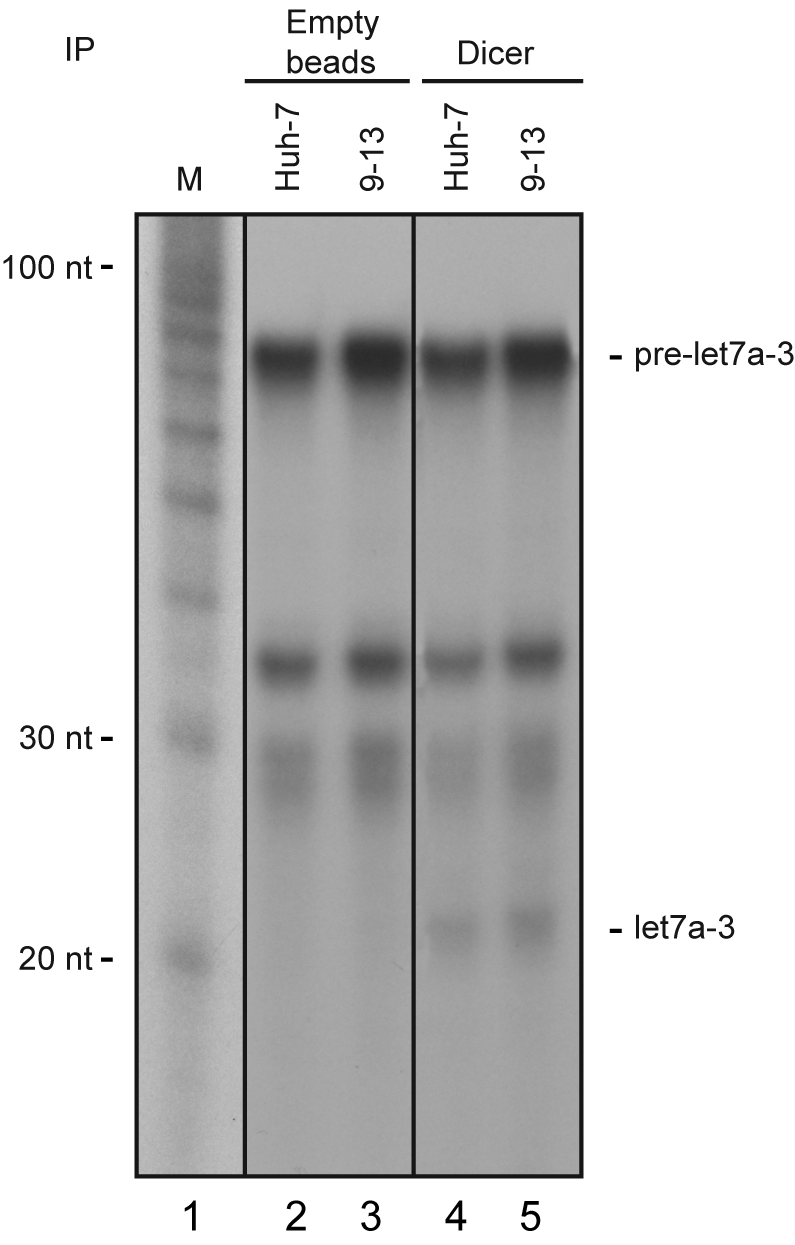

Supplement: Additional file 2 — Dicer in functionally competent in Huh-7 and 9–13 cells. The data provided indicate that the activity of Dicer is not influenced by HCV in vivo. The human pre-let7a-3 RNA was transcribed and randomly labeled (α-32P UTP, Perkin Elmer) by in vitro transcription using T7 promoter (Ambion) and purified by 10% denaturating PAGE. Huh-7 and 9–13 cells were resuspended in lysis buffer (Tris·HCl 50 mM, 137 mM NaCl, Triton X-100 1%) and immunoprecipitation (IP) was performed on 1 mg of proteins incubated with protein-G beads alone or beads/rabbit anti-Dicer at 4°C for 3 hours. Immune complexes were washed 3 times in lysis buffer, following by an additional wash in Tris·HCl 20 mM and MgCl2 2 mM, pH 7.5. α-32P labeled pre-let7a-3 RNA was incubated with immune complexes for in vitro processing of pre-miRNA in Dicer RNase activity assay for 1 hour at 37°C in Tris·HCl 20 mM, DTT 1 mM, ATP 1 mM, MgCl2 5 mM and 5% SUPERase∙In (Ambion), pH 7.5. Proteins were extracted by phenol/chloroform and RNA was precipitated and analyzed by denaturating PAGE and autoradiography. [file 1477-5751-8-8-S2.tiff]
